# *Toxocara* Infection in Psychiatric Inpatients: A Case Control Seroprevalence Study

**DOI:** 10.1371/journal.pone.0062606

**Published:** 2013-04-23

**Authors:** Cosme Alvarado-Esquivel

**Affiliations:** Department of Infectology, Faculty of Medicine and Nutrition, Juárez University of Durango State, Durango, Mexico; National University of Singapore, Singapore

## Abstract

**Background:**

There is poor knowledge about the epidemiology of toxocariasis in psychiatric patients.

**Aims:**

Determine the seroepidemiology of *Toxocara* infection in psychiatric patients.

**Methods:**

Through a case-control seroprevalence study, 128 psychiatric inpatients and 276 control subjects were compared for the presence of anti-*Toxocara* IgG antibodies in Durango, Mexico. Socio-demographic, clinical, and behavioral characteristics of inpatients associated with toxocariasis were also investigated.

**Results:**

Six of the 128 (4.7%) psychiatric inpatients, and 3 (1.1%) of the 276 controls were positive for anti-*Toxocara* IgG antibodies (*P* = 0.03). Stratification by age showed that *Toxocara* seroprevalence was significantly (*P* = 0.02) higher in patients aged ≤50 years old (6/90∶6.7%) than controls of the same age (2/163∶1.2%). While *Toxocara* seroprevalence was similar in patients and controls aged >50 years old. Stratification by gender showed that *Toxocara* seroprevalence was significantly (*P* = 0.03) higher in female patients (2/37∶5.4%) than in female controls (0/166∶0%). No statistically significant associations between *Toxocara* seropositivity and clinical characteristics were found. In contrast, *Toxocara* seropositivity was associated with consumption of goat meat and raw sea snail.

**Conclusions:**

This is the first report of toxocariasis in psychiatric inpatients in Mexico. Further studies with larger sample sizes are needed to elucidate the association of toxocariasis with psychiatric diseases. The role of the consumption of goat meat and raw sea snail in the transmission of *Toxocara* deserve further investigation.

## Introduction

*Toxocara* is a parasite widely distributed around the world [1], [2]. *Toxocara* eggs are shed by infected dogs and cats that contaminate the local environment [3], [4]. If embryonated eggs are accidentally ingested by humans, larvae hatch and disseminate via the bloodstream to anywhere in the body including liver, lungs, muscles, eyes, and central nervous system [2], [5]. Infection with *Toxocara* may also occur by ingesting *Toxocara* larvae from undercooked giblets [6]. Infections with *Toxocara* are usually asymptomatic; however, some infected individuals may develop a serious illness and death [1], [2], [7]. Many patients with ocular toxocariasis suffer from permanent vision loss [8]. The global impact of human toxocariasis is poorly understood [9]. There is very little knowledge about the epidemiology of *Toxocara* infection in psychiatric patients. Therefore, this study was aimed to determine the seroprevalence of *Toxocara* infection in psychiatric inpatients in Durango, Mexico. In addition, socio-demographic, clinical, and behavioral characteristics of psychiatric inpatients associated with toxocariasis were investigated.

## Methods

Through a case-control study using serum samples from recent *Toxoplasma gondii* serosurveys [10], [11], 129 psychiatric patients and 276 control subjects were compared for the presence of anti-*Toxocara* IgG antibodies. Inclusion criteria for the psychiatric patients were: 1) psychiatric inpatients of the Hospital of Mental Health “Dr. Miguel Vallebueno”; 2) aged 16 years and older; and 3) who accepted to participate in the study. Psychiatric patients included 39 females and 89 males aged 16–83 years old (mean 43.57+/−14.48 years). Patients suffered from organic, including symptomatic, mental disorders (F00–09) (n = 27), mental and behavioral disorders due to psychoactive substance use (F10–19) (n = 32), schizophrenia, schizotypal and delusional disorders (F20–29) (n = 39), mood (affective) disorders (F30–39) (n = 7), neurotic, stress-related and somatoform disorders (F40–49) (n = 3), mental retardation (F70–79) (n = 17), and epilepsy (G40) (n = 3). Control subjects consisted of 166 females and 110 males aged 16–91 years old (mean 45.45+/−18.06 years).

This study was approved by the Ethical Committee of the Instituto de Seguridad y Servicios Sociales de los Trabajadores del Estado in Durango City. Only archival serum samples and questionnaires from previous surveys [10], [11] were used in the present study. However, in the previous surveys, the purpose and procedures of the studies were explained to all participants. In addition, a written informed consent was obtained from all participants and from the next of kin of minor participants. The capacity to consent in psychiatric patients was determined through a clinical evaluation by hospital psychiatrists. Only psychiatric patients with capacity to consent and who accepted to participate were included in the study. Both previous surveys were approved by Institutional Ethical Committees.

The characteristics of the participants were obtained by using a standardized questionnaire. Socio-demographic data including age, gender, occupation, birth place, residence, educational level, and socioeconomic level were obtained from all participants. Clinical data explored included the presence of underlying diseases, memory, reflex, hearing, and visual impairments, and history of blood transfusion or transplants. Behavioral data included animal contacts, traveling, meat consumption (pork, beef, goat, mutton, boar, chicken, turkey, rabbit, venison, squirrel, horse, or other), consumption of raw or undercooked meat, unpasteurized milk, dried or cured meat (ham, sausages, salami or chorizo), consumption of unwashed raw vegetables, fruits, or untreated water, frequency of eating out of home (in restaurants or fast food outlets), contact with soil (gardening or agriculture), and types of floors at home from all participants were obtained.

Serum samples were obtained from all participants and kept frozen at –20°C until analyzed. Serum samples were analyzed for anti-*Toxocara* IgG antibodies with a commercially available enzyme immunoassay “*Toxocara”* kit (Diagnostic Automation, Inc. Calabasas, CA, U.S.A.). Absorbance reading equal to or greater than 0.3 OD units was considered positive. All tests were performed following the instructions of the manufacturer.

The statistical analysis was performed with the aid of the software Epi Info version 3.5.3 and SPSS version 15.0. The Pearson’s chi-square test and the Fisher exact test (when values were less than 5) were used for comparison of the frequencies among groups. Bivariate and multivariate analyses were used to assess the association between the characteristics of patients and *Toxocara* seropositivity. Variables were included in the multivariate analysis if they had a *P* value equal to or less than 0.20 in the bivariate analysis. Multivariate analysis was performed using multiple, unconditional logistic regression. A *P* value less than 0.05 was considered statistically significant.

## Results

Six of the 128 (4.7%) psychiatric inpatients, and 3 (1.1%) of the 276 controls were positive for anti-*Toxocara* IgG antibodies. The difference in the seroprevalences between the groups was statistically significant (*P* = 0.03). Stratification by age showed that *Toxocara* seroprevalence was significantly (*P* = 0.02) higher in patients aged ≤50 years old (6/90∶6.7%) than controls of the same age (2/163∶1.2%). While *Toxocara* seroprevalence was similar (*P* = 0.74) in patients >50 years old (0/38∶0%) than controls of the same age (1/113∶0.8%). Stratification by gender showed that *Toxocara* seroprevalence was significantly (*P* = 0.03) higher in female patients (2/37∶5.4%) than in female controls (0/166∶0%). No statistically significant (*P* = 0.38) difference in the *Toxocara* seroprevalence between male patients (4/89∶4.5%) and male controls (3/110∶2.7%) was found. Bivariate analysis of general socio-demographic characteristics in inpatients including age, gender, occupation, birth place, residence, educational level, and socioeconomic level did not show an association with *Toxocara* seropositivity.

With respect to clinical data, no statistically significant association between *Toxocara* seropositivity and psychiatric diagnosis was found. Of the 6 seropositive patients, one suffered from mental disorder (F06.8), three from mental and behavioral disorders due to psychoactive substance use (F10–19), one from schizophrenia (F20.3), and one from mental retardation (F72). No statistically significant associations between *Toxocara* seropositivity and other clinical characteristics including presence of underlying diseases, memory, reflex, hearing, and visual impairments, and history of blood transfusion or transplants were found.

As to behavioral data, seroprevalence of *Toxocara* seropositivity was significantly (*P* = 0.01) higher in patients with consumption of goat meat (3/24∶12.5%) than those without this behavioral characteristic (0/67∶0%). Similarly, seroprevalence of *Toxocara* seropositivity was significantly (*P* = 0.04) higher in patients with consumption of raw sea snail (1/1∶100%) than those without this behavioral characteristic (5/127∶3.9%). Other behavioral characteristics including animal contacts, traveling, consumption of other types of meat, consumption of raw or undercooked meat, unpasteurized milk, unwashed raw vegetables and fruits, or untreated water, frequency of eating out of home (in restaurants or fast food outlets), contact with soil (gardening or agriculture), and types of floors at home were observed in similar frequencies among seropositive and seronegative patients (*P≥*0.05). Multivariate analysis of behavioral variables with *P*<0.20 obtained in the bivariate analysis did not show any significant association.

## Discussion

In the present study, a significantly higher seroprevalence of *Toxocara* seropositivity in psychiatric inpatients than in control subjects was found. Furthermore, comparison with stratification by gender and age among cases and controls showed an increased *Toxocara* seroprevalence in female inpatients and in those aged ≤50 years old. To the best of my knowledge there are not further case control studies of toxocariasis in psychiatric inpatients. However, the increased *Toxocara* seroprevalence found in the present study agrees with that found in a descriptive study in Sicily, Italy [12], where researchers found the highest seroprevalence of *Toxocara* antibodies in psychiatric patients (13%) as compared with farmers (9.4%), children (1.9%), and blood donors (1.2%). It is not clear why psychiatric inpatients had an increased *Toxocara* seroprevalence. However, it is likely that some epidemiological factors in inpatients might have contributed for infection. Poor hygiene practices were observed in the psychiatric inpatients and this factor might have contributed for an increased *Toxocara* exposure. Similarly, many inpatients have had contact with cats and dogs. In a local context, the seroprevalence found in psychiatric inpatients is lower than the 13% and 26.2% seroprevalences of *Toxocara* exposure found in waste pickers [13] and persons of Tepehuanos ethnicity [14] in Durango, Mexico, respectively. Low sanitation and poor hygiene practices are common among the 3 groups of population. Remarkably, bivariate analysis showed associations of *Toxocara* seropositivity with consumption of goat meat and raw sea snail. I am not aware of any report about such associations. However, the epidemiological link of consumption of goat meat with toxocariasis is possible. Finsterer and coworkers [15] reported a case of neurotoxocariasis with anti-*Toxocara* antibodies in serum and cerebrospinal fluid associated with lower motor neuron disease in a man who habitually ate raw goat meat. Therefore, the association of toxocariasis and consumption of goat meat warrant for further research.

In the present study, only few *Toxocara* seropositive inpatients were found. This fact was certainly a limitation of the study that avoided reaching further statistically significant associations between *Toxocara* seropositivity and socio-demographic, clinical and behavioral characteristics. The low number of *Toxocara* seropositive inpatients also avoided to obtain any association of toxocariasis with behavioral characteristics by multivariate analysis. Further research with a larger sample size should be conducted to investigate the association of toxocariasis with socio-demographic, clinical and behavioral characteristics of psychiatric inpatients.

At present, there is scanty information about a relationship of toxocariasis with psychiatric diseases. In a Turkish study, *Toxocara* seroprevalence was found higher in patients suffering from schizophrenia (45.9%) than in control subjects (2%) [16]. In Germany, Richartz and Buchkremer [17] reported a case of a woman with cerebral toxocariasis with depressive symptoms and cognitive deficits. In the present study, there was no association of toxocariasis with schizophrenia, depression or cognitive deficits. However, the low frequency of seropositive patients and the low number of patients with schizophrenia, depression and cognitive disorders in the present study did not allow to support or challenge such associations. Further research with a larger sample size should be conducted to investigate the association of toxocariasis with specific psychiatric diseases including schizophrenia, and depressive and cognitive disorders. The higher seroprevalence of *Toxocara* seropositivity in psychiatric inpatients than in control subjects found in the present study may means: a) a causal relationship between infection and psychiatric diseases; b) an increased risk of acquiring infection in psychiatric patients due to behavioral changes; or c) any other reason. An infection acquired in the hospital cannot be excluded. In fact, a cat and a dog were observed in the psychiatric hospital. It would be of interest to study the association of *Toxocara* infection and psychiatric disease in outpatients, and in acute and chronic psychiatric patients.

This is the first report of toxocariasis in psychiatric inpatients in Mexico. Further studies with larger sample sizes are needed to elucidate the association of toxocariasis with psychiatric diseases. The role of the consumption of goat meat and raw sea snail in the transmission of *Toxocara* deserve further investigation.
